# Continuous non-locking vs. interrupted suturing techniques for the repair of episiotomy or second-degree perineal tears: A single-blind randomized controlled trial

**DOI:** 10.3389/fsurg.2023.1114477

**Published:** 2023-04-05

**Authors:** Shahla Faal Siahkal, Parvin Abedi, Mina Iravani, Parvin Esfandiarinezhad, Maryam Dastoorpoor, Shahla Bakhtiari, Mahin Najafian, Foruzan Sharifipour, Zaynab Mohaghegh

**Affiliations:** ^1^ Department of Midwifery, School of Nursing and Midwifery, Ahvaz Jundishapur University of Medical Sciences, Ahvaz, Iran; ^2^ Department of Midwifery, Marand Branch, Islamic Azad University, Marand, Iran; ^3^ Department of Midwifery, Menopause Andropause Research Centre, Ahvaz Jundishapur University of Medical Sciences, Ahvaz, Iran; ^4^ Reproductive Health Promotion Research Center, Midwifery Department, Nursing and Midwifery School, Ahvaz Jundishapur University of Medical Sciences, Ahvaz, Iran; ^5^ Department of Epidemiology and Biostatistics, Menopause Andropause Research Center, Ahvaz Jundishapur University of Medical Sciences, Ahvaz, Iran; ^6^ Midwifery Department, Rosie Hospital, Cambridge University Hospitals NHS Foundation Trust, Cambridge, United Kingdom; ^7^ Department of Obstetrics and Gynecology, School of Medicine, Fertility, Infertility and Perinatology Research Center, Ahvaz Jundishapur University of Medical Sciences, Ahvaz, Iran; ^8^ Department of Midwifery, School of Nursing and Midwifery, Kermanshah University of Medical Sciences, Kermanshah, Iran

**Keywords:** episiotomy, perineal pain, wound healing, continuous non-locking suturing technique, interrupted suturing technique

## Abstract

**Objective:**

Perineal trauma is a serious and frequent problem after childbirth which is experienced by millions of women worldwide. The technique used for perineal repair may have an impact on pain and wound healing. The aim of the present study was to compare the continuous non-locking technique with interrupted suturing for the repair of episiotomy or second-degree perineal tears.

**Methods:**

A single-blind randomized-controlled trial was conducted from October 2021 to August 2022 in Sina Hospital, Ahvaz, Iran. Three hundred women were selected and randomly assigned into control and intervention groups using block randomization technique. The main outcomes included pain and wound healing that were assessed using visual analog scale (VAS), as well as redness, edema, ecchymosis/bruising, discharge, and approximation scale (REEDA). The secondary outcomes were the use of analgesics, duration of perineal repair, material used for suturing, pain during urination and defecation, and resumption of sexual intercourse. The participants were followed up on the first and seventh days and in the 6th week postpartum. Mann-Whitney, Chi-square, and Generalized Estimating Equations (GEE) model were used for data analysis.

**Results:**

Wound healing was significantly better in the continuous non-locking suture technique compared to the interrupted technique (*β* = −1.98; *P* > 0.0001). Women also experienced less pain in the continuous non-locking suture technique (*β *= −2.46; *P* > 0.0001). There was a reduction in the use of analgesics, the duration of perineal repair, and the material used for suturing in the continuous non-locking suturing technique as opposed to the interrupted method (*P* < 0.0001).The odds of pain during urination and defecation significantly reduced in women who underwent the continuous non-locking method (*P* < 0.001). Also, women in the continuous non-locking group resumed their sexual intercourse earlier (*P* < 0.0001).

**Conclusion:**

The findings of this study revealed that use of continuous non-locking technique for suturing was associated with reduced perineal pain and improved wound healing. Furthermore, it was associated with a shorter duration of perineal repair, less suture material used, and less need for analgesics compared with the interrupted method. There is, however, need for more studies to confirm the results of the present study.

Iranian registry for randomized controlled trials (Ref. ID: IRCT20190415043283N1).

## Background

Perineal trauma during childbirth affects millions of women worldwide and can lead to long-term maternal complications (1). Perineal trauma may occur spontaneously during childbirth or as a result of a surgical incision made by an obstetrician or a midwife (episiotomy) to increase the diameter of the vaginal outlet to facilitate childbirth (2). According to the World Health Organization (WHO), the incidence of perineal tears in 2015 was 2.7 million cases, which is estimated to reach 6.3 million in 2050 in the world, with 50% of these cases occurring in Asia (3). In Iran, the actual rate of episiotomies is unknown, but according to the available data, it has been reported to range from 41.5% to 97% (4). The prevalence of perineal trauma varies considerably with individual practices and the policies of every institution throughout the world (5). The majority of women experience some short-term complications such as pain after perineal repair, but up to 20% will continue to have long-term problems such as sexual discomfort (6). This injury can also cause urinary or anal incontinence (7).

Pain is a common problem associated with episiotomy. The prevalence of postpartum perineal pain at the first day after delivery is estimated to be 88.2% which falls to 62.3% at the end of the first week after birth (8). Perineal pain can affect the physical, psychological, and social well-being of women (9). Problems such as failure to do daily activity, lag in breastfeeding, deterioration of maternal–infant attachment, and failure to fulfill maternal roles have been reported as short-term effects of perineal pain (6). In addition to the extent of the tear, operative skills, the type of material used, and the suturing techniques can also have an important effect on the pain and discomfort after repair (7).

Different studies have shown that suturing techniques and materials are associated with different complications and pathologies in women after childbirth (6, 7, 10).

The best technique for repairing perineal tears or episiotomy is the technique that requires less time, using less materials, and causing less pain in the short- and long-term, allowing the resumption of intercourse earlier and requiring less suture removal and re-suturing (11). There are different techniques to repair perineal tears, namely interrupted suture, continuous with locking suture, and continuous non-locking suture (1). The most commonly used technique is the interrupted suture in which the perineal skin is closed with separate stitches (7). In continuous non-locking technique for a vaginal trauma, the deep tissues and mucosa are closed with a single continuous non-locking stitch. In this method, the perineal muscles are also made closer to each other using a similar continuous non-locking technique, and the repair is completed with a continuous suture inserted well below the skin surface in the subcutaneous fascia (1). A large-scale RCT involving 1,542 women compared the effect of using continuous non-locking and interrupted techniques for episiotomy and second-degree perineal repair. The results showed that the continuous non-locking sutures significantly reduced perineal pain from 10 days up to 12 months after childbirth (12). In contrast, Valenzuela et al. found that pain on the second and 10th postpartum day, and 3 months postpartum was not different between interrupted and non-locking continuous sutures (13). In Iran, perineal trauma is traditionally repaired using the interrupted technique, and midwives are responsible for suturing the majority of second-degree perineal tears and episiotomies sustained during spontaneous vaginal delivery. To the best of our knowledge, the effect of continuous non-locking technique has not been investigated so far in Iran. Considering the high prevalence of episiotomy in Iran, the primary aim of this study was to compare pain and wound healing in non-locking and interrupted techniques. The secondary objective was comparison of the use of analgesics, duration of perineal repair, the material used for suturing, pain during urination and defecation, and resumption of sexual intercourse between the two groups.

## Methods

### Setting and participants

This was a randomized controlled trial performed on primiparous and multiparous women referring to Sina Hospital in Ahvaz, Iran, from October 2021 to August 2022. Sina Hospital is a secondary care university hospital affiliated to Ahvaz Jundishapur University of Medical Sciences. The number of births in this hospital in 2021 was 4,496, of which 64.45% were vaginal births. The percentage of episiotomy in this hospital is reported to be 37.66%, and about 70% of mothers were primiparous.

Women were eligible to participate in this study if they: were literate, had singleton pregnancy with cephalic presentation, had normal body mass index (BMI = 19.5–24.9), were at full-term gestational age (37–41 weeks), had normal neonate weight, had undergone mediolateral episiotomy or second-degree perineal tear, and were willing to participate in this study. Exclusion criteria included women with AIDS (acquired immunodeficiency syndrome), hepatitis B virus infection, severe perineal warts, extensive varicose veins of the external genitalia, 3rd or 4th degree of perineal tears, and no access to a phone line (for follow-up). All participants were carefully assessed through taking a complete history and conducting thorough examination to ensure that the inclusion criteria were met.

### Sample size

The sample size was calculated based on the prevalence of pain 10 days after childbirth and based on a previous study by Kettle et al. (12). We considered *p*_1_ = 0.2600, *p*_2_ = 0.4400, *α* = 0.05, and power = 95%. We added 10% for drop-outs, and the final sample size was calculated to be 136. The final sample size was set to be 150, assuming a loss to follow-up of 10%.

$$n=\frac{{\left(z_{1-\alpha /2}+z_{1-\beta }\right)}^{2}\;*\;[\;p_{1}(1-p_{1})+p_{2}(1-p_{2})]}{{\left(p_{1-}p_{2}\right)}^{2}}$$

### Sampling

After receiving approval from the Ethics Committee of Ahvaz Jundishapur University of Medical Sciences (Ref. ID: IR. AJUMS.REC.1398.019), the lead researcher attended the maternity ward of Sina Hospital. Eligible women were identified, and if they were willing to participate in the study, the written informed consent was obtained.

### Randomization

Three hundred primiparous and multiparous women who had a vaginal delivery with a second-degree perineal tear or episiotomy were selected and randomly assigned into two groups of non-locking continuous or interrupted suturing techniques using block randomization with a block size of 4 and an allocation ratio of 1 : 1. Randomization was performed using codes generated by Epi Info software version 6 (Epi Info™, Centers for 131 Disease Control and Prevention, Atlanta, GA, United States). For allocation concealment, the type of intervention was written in a piece of paper and was placed inside consecutively numbered opaque envelopes which were kept by a person who was not aware of the objectives of the study. Therefore, neither the researchers nor the participants were aware of grouping until the commencement of the study. After informed consent was obtained from eligible women, the envelopes were opened, and the intervention started. The allocation sequence was determined by a person who was neither involved in the sampling and data collection nor aware of the study process. Due to the nature of the study, it was not possible to blind the researchers; however, the participants and the data analyzer were blinded to grouping.

It should be noted that childbirth process was managed by the lead researcher. In both groups, repair of episiotomy and second-degree tears was done by the lead researcher herself and her assistant. The researchers that placed the sutures had previously participated in a workshop on continuous non-locking suturing technique by lead midwife in the delivery unit at Rosie Hospital, Cambridge University Hospitals NHS Foundation Trust.

### Intervention

After assigning participants into study groups, the researchers administered the infiltration of the local analgesia along with the episiotomy 2 min before suturing. In both groups, 2 min before episiotomy, 5 ml of 2% lidocaine was injected in the incision points at 7, 8, and 9 o'clock. Then, 2 min before starting to repair the episiotomy, depending on the size of tear and in case the mother cannot tolerate the pain, 2–5 ml of 2% lidocaine was injected at the site of episiotomy incision.

Then perineal repair was done by one of the following techniques. The continuous non-locking method was done in 3 steps: Step-1(suturing the vaginal wall), Step-2 (suturing the perineal muscle layer), and Step-3 (suturing the skin layer). Step-1: First, the apex of the vaginal trauma was identified (A). Then the first stitch was placed 5 mm–10 mm above the apex to secure any bleeding points that may not have been visible (B). The first stitch was secured using a surgeon's square knot (cutting off the short end of the suture material, leaving about 1 cm–2 cm) (C). Continuous non-locking stitches were used for the repair of vaginal wound (usually about three to four stitches) until hymenal remnants (D). Sutures were placed approximately 5 mm to 10 mm from wound edges, and each stitch reached the trough of the wound to close any dead space. One suture was inserted to close the hymenal ring (E). Step-2: The needle was inserted at the level of the fourchette (near to the hymenal ring) to emerge deep in the center of the muscle layer (F). The depth of the trauma was checked (G). The continuous non-locking suturing technique was maintained, and each stitch was placed 5 mm–10 mm below the wound skin edges, with each stitch being matched for both depth and width (H). The perineal muscles were closed in one layer, or in case of deep trauma, two layers were used (I). Finally, the needle was removed from the inferior part of the trauma (J).

Step-3: The stitching direction was reversed at the inferior part of the trauma (K). The perineal skin was closed by inserting fairly deep sutures in the subcutaneous layer (L). Stitches were placed opposite each other, when they were not pulled too tight and approximately 5 mm–10 mm apart (M). Then, the needle was swung under the tissue into the vagina behind the hymenal remnants, and the repair of the hymenal ring was completed (N). The repair was completed using an Aberdeen knot (O).

The wound of the other group was repaired using the interrupted suturing method as follows: The first stitch was inserted above the apex of vaginal trauma to secure any bleeding points (A). Vaginal trauma was closed using continuous locking stitches (B), and tied at the fourchette with a loop knot (C). Interrupted sutures were inserted to close the perineal muscles (deep and superficial) (D), and interrupted transcutaneous stitches were inserted near the skin edges (E).

All episiotomy or perineal tears were repaired while the women were in the lithotomy position. For all women, standard polyglactin 910 suture thread was used for suturing with taper point ½ circle 30 mm, and 75 cm violet (Smi, Belgium). Diclofenac suppositories and ibuprofen tablets were used for pain relief up to 3 days after childbirth.

After childbirth, the necessary training on perineal hygiene was provided for both groups. The women were followed on the first, seventh, and 40th days postpartum. During the follow-ups, the phone number of researchers was made available to the women so that they could contact in case they had any question. During follow-up visits, the women were examined in the lithotomy position in the obstetrics clinic of Sina Hospital, and the episiotomy wound healing and pain intensity were evaluated using the episiotomy healing assessment scale: Redness, Oedema, Ecchymosis, Discharge, Approximation (REEDA) and the VAS score.

### Data collection tools

A questionnaire for socio-demographic and obstetric characteristics, VAS, and REEDA scale were used to collect the data. The questionnaire for socio-demographic and obstetric characteristics included questions about age, weight, gravidity, parity, gestational age, weight of the neonate, cervical dilatation at admission, interval between rupture of membrane and delivery, the duration of the first, second and third stages of labor, frequency of vaginal exams, time needed for the repair of episiotomy, number of suture packets used, lidocaine administration, pain during urination and defecation, and the use of analgesic pills. These were assessed by the observer researcher through examination and were recorded in the questionnaire.

VAS is a validated scale of 10 cm with zero indicating no pain and 10 indicating maximum imaginable pain (14). The patients were asked to mark their pain on the VAS scale (15). This scale is widely used in other studies, and its reliability and validity have already been confirmed (ICC = 0.99, and Spearman's *r* coefficient *=* 0.818), respectively (14, 16). In Iran, the reliability of this scale has been confirmed by obtaining a correlation coefficient of *r* = 0.88 in Rezvani et al. (2012) (17).

The REEDA scale is an instrument for measuring perineal healing. This scale consists of five criteria including redness, edema, ecchymosis, discharge, and approximation. Each item is scored between 0 and 3, and the total score is 15. The sum of the scores represents the overall healing score, with smaller scores showing better perineal healing (18). The reliability and validity of this instrument have been confirmed in Alvarenga et al. (2015) (19). In Iran, the reliability and validity of REEDA scale were confirmed by Pazandeh et al. (20).

### Data analysis

Data analysis was done using SPSS version 22. Mean and SD, or number and frequency were used to report the descriptive data. Shapiro–Wilk test was used to evaluate the normality of the data. Independent *t*-test or Mann–Whitney-*U* test was used to analyze continuous data. The Chi-square test was used for categorical data. Also, the Generalized Estimating Equations (GEE) test was used to compare the average VAS score and REEDA at three time points (follow ups). All analyses were done assuming an alpha less than 0.05% and 95% confidence interval to set the significance level.

## Results

### Recruitment and follow-up

**Figure 1:**
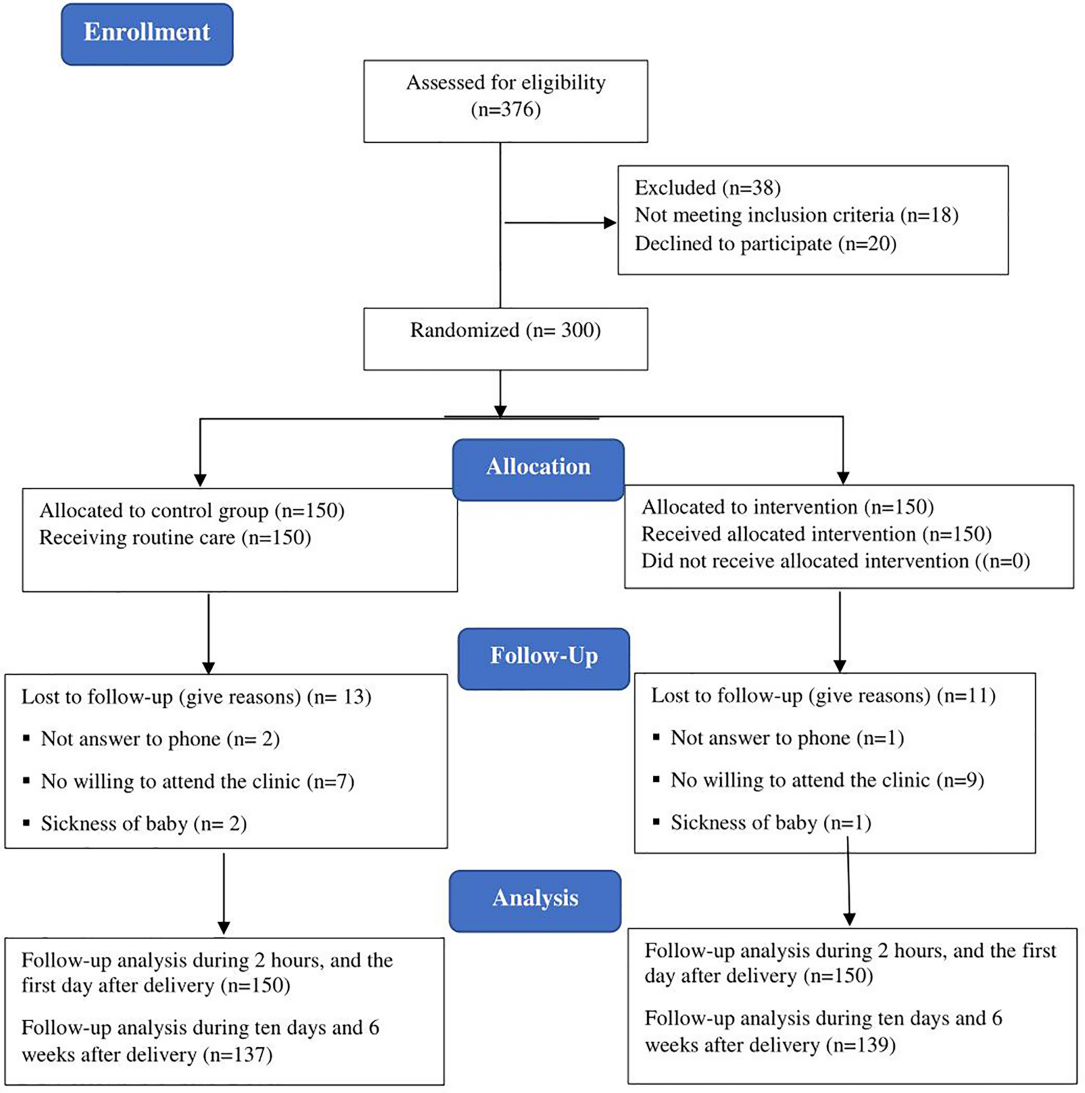
Flow diagram of the participants through the study.

Between April 2021 and December 2021, 300 eligible women were enrolled in this study. Of these women, 11 (7.3%) in the intervention group and 13 (8.6%) in the control group were not willing to continue participation for check-ups on the 10th and 42nd days after childbirth due to high workload. The data of these women were analyzed until the first day after childbirth. Data related to the remaining 276 women (139 women in the intervention group and 137 in the control group) were included in our final analyses. Figure 1 shows the flow chart of the study.

### Participant characteristics

The demographic characteristics of the recruited women were described are shown in Table 1. The mean ± SD age of women in the intervention group was 25.31 ± 6.50 compared to 25.62 ± 6.16 years in the control group (*P* = 0.72). The majority of women in intervention and control groups were housewives (98.00% and 90.00%, respectively).

**Table 1:** The sociodemographic and obstetric characteristics of participants in two groups of non-locking suturing technique and interrupted technique.

Characteristics		Non-locking suturing technique (*n *=* *150)	Interrupted technique (*n* = 150)	*P* value
		Mean ± SD		
Age (year)		25.31 ± 6.50	25.62 ± 6.16	*0.72
Weight (kg)		75.68 ± 12.66	73.46 ± 11.59	*0.27
Gestational age (week)		39.19 ± 1.02	39.00 ± 1.17	*0.16
Birth weight (gr)		3,203.84 ± 445.32	3,248.90 ± 598.68	*0.07
Head circumference (cm)		34.12 ± 1.54	34.10 ± 1.50	*0.63
1st min. Apgar		8.98 ± 0.18	9.00 ± 0.00	*0.15
5th min. Apgar		10.00 ± 0.00	10.00 ± 0.00	a
Cervical dilatation in admission (cm)		4.08 ± 1.97	4.42 ± 2.38	*0.33
Interval between ROM^a^ to delivery (min)		74.36 ± 129.18	117.93 ± 148.54	*0.000
Duration of the first stage of labor (min)		351.38 ± 190.4	330.03 ± 190.7	*0.14
Duration of the second stage of labor (min)		27.92 ± 17.46	27.83 ± 18.20	*0.69
Duration of the third stage of labor (min)		6.37 ± 7.16	6.77 ± 5.43	*0.15
Number of vaginal exams		10.05 ± 2.78	11.20 ± 8.13	*0.11
Time for repair of perineal tears (min)		17.02 ± 5.10	24.32 ± 10.83	*<0.0001
Number of suture packets used		1.07 ± 0.26	1.90 ± 0.45	*<0.0001
Use of lidocaine (ml)		5.04 ± 1.00	7.30 ± 3.03	*<0.0001
Perineal status		*N* (%)	*N* (%)	
Episiotomy		95 (63.3)	121 (80.7)	***0.001
2nd degree laceration		55 (36.7)	29 (19.3)	***0.001
Gravida	1	62 (41.3)	64 (42.7)	***0.81
	≥2	88 (58.7)	86 (57.3)	
Parity	1	116 (77.3)	82 (54.7)	***<0.0001
	≥2	34 (22.7)	68 (45.3)	
Education	Primary	87 (58)	92 (61.3)	***0.171
	High school or diploma	33 (22)	21 (14)	
	University degree	30 (20)	37 (24.7)	
Occupation	House wife	147 (98.00)	135 (90.00)	***0.31
	Employed	3 (2.00)	15 (10.00)	
Economic situation	Poor	11 (7.3)	20 (13.3)	***0.23
	Medium	138 (92.00)	129 (86.00)	
	Good	1 (0.7)	1 (0.7)	
Induction of labor		33 (22.00)	52 (34.7)	***<0.0001
Rapture of membrane				
Spontaneous		107 (71.3)	103 (68.7)	***0.61
Artificial		43 (28.7)	47 (31.3)	
Sexual activity within 42 days after birth		60(43.2)	17(12.4)	***0.0001

^a^ Rupture of membrane.

* Mann–Whitney *U* test.

** Independent *t*-test.

*** Chi square test.

a *t* cannot be computed because the standard deviations of both groups are 0.

The two groups were similar in terms of gestational age, educational attainment, occupation, gravidity, cervical dilatation upon admission to hospital, frequency of vaginal examinations, the interval between rupture of membranes and birth, birth weight, head circumference, and Apgar scores. There was no statistically significant difference between the intervention and control groups regarding the length of the first, second, and third stages of labor (*P* > 0.05) Table 1.

### Primary outcomes

Mean pain intensity at baseline was 2.74 ± 1.48 in the intervention group and 6.28 ± 2.38 in the control group. At all three follow-up assessments, intensity of pain in the intervention group was significantly lower than that in the control group. Mean baseline REEDA score was 0.24 ±** **52 and 3.50 ±** **2.50 in the intervention and control groups, respectively. The REEDA score of the intervention group was significantly lower than that in the control group at all three follow-up assessments (*P* < 0.05) (Table 2).

**Table 2:** Mean of visual analogue scale and redness, oedema, ecchymosis, discharge, approximation scale variables in the intervention and control groups.

Variable	Time	Group	Mean ± SD	*P* value
REEDA^a^	First day	Non-locking suturing technique	0.24 ± 0.52	*0.000
		Interrupted suturing technique	3.50 ± 2.50	*0.000
REEDA	10^th^ day	Non-locking suturing technique	0.15 ± 0.46	*0.000
		Interrupted suturing technique	2.37 ± 1.42	*0.000
REEDA	6 weeks	Non-locking suturing technique	0.04 ± 0.20	*0.000
		Interrupted suturing technique	0.75 ± 0.89	*0.000
VAS^b^	2 h	Non-locking suturing technique	2.74 ± 1.48	*0.000
		Interrupted suturing technique	6.28 ± 2.38	*0.000
VAS	First day	Non-locking suturing technique	0.71 ± 0.86	*0.000
		Interrupted suturing technique	4.6 2 ± 2.08	*0.000
VAS	10^th^ day	Non-locking suturing technique	0.51 ± 1.03	*0.000
		Interrupted suturing technique	2.68 ± 1.77	*0.000
VAS	6 weeks	Non-locking suturing technique	0.38 ± 0.97	*0.000
		Interrupted suturing technique	0.79 ± 1. 10	*0.000

^a^ Redness, Oedema, Ecchymosis, Discharge, Approximation.

^b^ Visual Analogue Scale.

* Mann–Whitney *U* test.

The results of the GEE model showed that using continuous non-locking technique has a statistically significant relationship with better wound healing in the intervention group compared to the control group. (*β* = −1.98, *P* < 0.0001). Furthermore, the results showed that the group with continuous non-locking technique experienced significantly less perineal pain (*β* = −2.46, *P* < 0.0001) (Table 3).

**Table 3:** Results of generalized estimating equations model in examining the trend of changes in variables of redness, oedema, ecchymosis, discharge, approximation scale and visual analogue scale in terms of groups and time.

Variable	Group/Time	*β*	SE	Wald Chi-Square	*P* value
REEDA^a^	Interrupted suturing technique (Baseline)	–	–	–	–
	Non-locking suturing technique	−1.98	0.11	279.30	0.000
	First day (Baseline)	–	–	–	–
	Tenth day	−0.64	0.08	58.78	0.000
	6 weeks	−1.42	0.11	144.56	0.000
VAS^b^	Interrupted suturing technique (Baseline)	–	–	–	–
	Non-locking suturing technique	−2.46	0.14	284.02	0.000
	2 h (Baseline)	–	–	–	–
	First day	−1.84	0.08	504.93	0.000
	Tenth day	−2.88	0.10	766.71	0.000
	6 weeks	−3.81	0.14	698.53	0.000

^a^ Redness, Oedema, Ecchymosis, Discharge, Approximation.

^b^ Visual Analogue Scale.

### Secondary outcomes

Using continuous non-locking technique significantly reduced the odds of pain during urination (OR = 0.07, 95% CI = 0.025–0.235, *P* < 0.001). Furthermore, the intervention group experienced less pain during defecation (OR = 0.04, 95% CI = 0.012–0.128, *P* < 0.001), and the used fewer analgesic pills (OR = 0.06, 95% CI = 0.041–0.099, *P* < 0.001, Table 4).

**Table 4:** Results of generalized estimating equations model in examining the trend of changes in secondary outcomes in terms of group and time.

Variable	Group/Time	*β*	SE	Wald Chi-Square	OR	*P* value
	Interrupted suturing technique (Baseline)	–	–	–	–	–
Urinary Pain	Non-locking suturing technique	−2.56	0.57	20.27	0.07	0.001
	2 h (Baseline)	–	–	–	–	–
	First day	−.16	0.087	3.71	.05	0.84
	Tenth day	−2.62	0.39	44.09	0.07	0.001
	6 weeks	−3.20	0.51	39.22	0.04	0.001
Defecation Pain	Interrupted suturing technique (Baseline)	–	–	–	–	–
	Non-locking suturing technique	−3.23	0.60	28.89	0.04	0.001
	2 h (Baseline)	–	–	–	–	–
	First day	−1.06	0.282	14.26	2.90	0.000
	Tenth day	.454	0.361	1.57	1.57	0.21
	6 weeks	−2.02	0.719	7.90	0.13	0.005
Use of analgesic pills	Interrupted suturing technique (Baseline)	–	–	–	–	–
	Non-locking suturing technique	−1.62	0.367	19.56	0.19	0.001
	2 h (Baseline)	–	–	–	–	–
	First day	0.73	0.39	3.45	2.082	.063
	Tenth day	1.61	0.42	14.81	5.040	0.001
	6 weeks	−0.13	0.53	0.067	0.871	0.79

The mean repair time of perineal lacerations in the continuous non-locking suture technique was significantly lower compared to the interrupted suture technique (17.02 ± 5.10 min vs. 24.32 ± 10.83, *P* < 0.0001). The mean number of suture packets used to perform the continuous non-locking technique was 1.07 ± 0.26 packets, compared with 1.90 ± 0.45 in the interrupted technique (*P* < 0.0001, Table 1).

Women in the intervention group resumed their sexual function earlier compared to the women in the control group (43.2% vs. 12.4%, *p* < 0.0001, Table 1).

## Discussion

This study compared the effects of continuous non-locking and interrupted techniques for suturing episiotomy or perineal tears on perineal healing and pain, pain during urination and defecation, resumption of coitus, and using analgesics.

The results of this study showed that using continuous non-locking technique for repair of perineal tears or episiotomy has beneficial effects on intensity of perineal pain and wound healing. According to the results, compared with the interrupted method, the continuous non-locking technique significantly reduced the level of perineal pain 2 h, 10 days, and 6 weeks after delivery. Also, our results indicated that the continuous suturing method can improve wound healing. Various studies have reported different results on the effect of continuous suturing method. Our findings are consistent with those of Besen et al. who reported that perineal pain was significantly lower in women treated with continuous suturing technique. They also found that this technique was more effective in fulfilling women's roles with respect to their daily life activities and neonatal care (6). In England, Kettle et al. reported a significantly lower intensity of perineal pain during 10 days postpartum in women with continuous technique than those undergoing the interrupted method. This difference persisted up to 12 months after delivery, but it was no longer statistically significant (12). Similarly, Martínez-Galiano et al. assessed pain intensity in 24 h, 15 days, and 3 months after delivery in two groups of primiparous women undergoing continuous and interrupted suturing. Their results showed that women who had a continuous suture repair had lower levels of pain from delivery up to 3 months after delivery (7). The main reason for the higher pain score and discomfort associated with the interrupted suturing technique is applying separate stitches to the perineal skin (12). Furthermore, in the interrupted method, the tension of the stitches is higher, which leads to edema and can increase pain. However, in the continuous method, the pressure is distributed along the whole length of a single suture and edema is not created. Another important factor for the less pain experienced in the continuous suture is that skin sutures are inserted into the subcutaneous tissue; thus, they do not stimulate nerve endings in the skin surface (1). These interpretations are consistent with our findings which show that continuous technique sutures were associated with lower postpartum pain and discomfort than interrupted technique.

Our results, however, are in contrast with those of Valenzuela et al. who found that pain on the second and 10th days, and in 3 months postpartum was not statistically different between the two techniques (13). This discrepancy is probably due to differences in the number of participants and the time of pain measurement. Kindberg et al. showed that there were no statistically differences between the two techniques in terms of perineal pain in 10 days postpartum (21). One reason justifying this inconsistency could be that in our study, the same person was responsible for the delivery and repair of the episiotomy or perineal tears, and the skill of the birth attendant and the type of material used were the same in both groups. However, in Kindberg et al., more than 70 midwives were involved, which will lead to great variation in surgical skills and clinical competence.

Several factors such as the type of suture material used, the suturing technique, and the skill of the healthcare professional have the greatest impact on the after-birth complications associated with perineal pain and wound healing (22). The Royal College of Obstetricians and Gynecologists (RCOG) guidelines recommend the use of continuous subcuticular technique for perineal skin closure and a loose, continuous non-locking suturing technique for vaginal tissue and perineal muscle as it is associated with less short-term pain compared with the interrupted method. This recommendation is also given level A gradation (23).

Our findings showed that wound healing was faster in the intervention group compared to the control group. In line with the results of the present study, a systematic review showed that the subcuticular technique was better than the interrupted transcutaneous technique for wound healing (24). Also, a study conducted by Besen et al. indicated that wound healing improved after application of continuous suture technique (24). However, Perveen et al. and Hasanpoor et al. reported no difference between the two techniques in terms of wound healing (25, 26). This discrepancy could be explained by the knots technique used. In the present study, we only applied two types of knots including square knot in the first and Aberdeen knot in the final stage of the perineal repair. We also used continuous non-locking sutures for repair of vaginal mucosa and perineal muscles. Evidence has shown that wound healing is a process that is related with perineal pain. In fact, the faster the wound healing, the faster the pain relief (27).

Our findings demonstrated that dysuria and defecation pain were statistically lower in the intervention group compared with the control group. However, since no previous study has analyzed this relationship, we were unable to compare these results with those of other studies. Also, there was no wound dehiscence or infection in both groups.

Our study found that the time taken to carry out perineal repair in the continuous suturing method was shorter than that spent on the interrupted suture. We also found that the amount of material used for the continuous suture technique was less compared with the interrupted suture technique. Furthermore, women in the intervention group took fewer analgesics. These findings are in agreement with other studies which demonstrated that continuous suturing needed shorter time and less material compared to interrupted suturing technique (6, 7, 11, 13, 22). This is because in the interrupted suturing technique, the stitches are individually tied, which needs more threads and longer repair time (28).

Our results revealed that women who had continuous sutures experienced less pain, so they took fewer analgesics.

Haydari et al. showed that 20% of women in Iran started sexual intercourse during the first month after vaginal delivery (29). The results of another study conducted in Southern Iran showed that only 29% of women who experienced vaginal delivery resumed their sexual relationship four weeks after childbirth (30). In the present study, the results showed that women in the intervention group resumed coitus after the 40th day postpartum on average. Various studies have reported different results regarding the effect of suturing method on sexual activity after childbirth. In a Turkish study, for example, Kokanali et al. showed that women started sexual intercourse 6 weeks after childbirth, but there was no statistically significant difference in the proportion of women who had started sexual intercourse (31). Besen et al. found that women in both groups started sexual activity 50 days after delivery on average (6). However, Perveen et al. (26) and Valenzuela et al. (13) reported opposite results, indicating a delayed re-initiation of sexual relations in the group receiving continuous sutures. This discrepancy could be due to sample size, pain evaluation intervals, and mothers' parity.

Results of a recent systematic review in Iran, involving 4,047 women reported that fear of damage to the perineum is one of the most common causes of fear of vaginal childbirth, and a factor contributing to the ever increasing rate of elective cesarean section (32). Given the alarmingly high rate of cesarean birth in Iran (33), approaches which can improve maternal outcomes and make positive experiences of childbirth for women, are top priority. Our results demonstrated that in addition to reducing perineal pain and promoting wound healing, continuous non-locking suturing required shorter time and less suturing material compared to interrupted suturing. These findings have particular implications for women and professionals since the reduced procedure time lowers the risk of infection, hemorrhage, and discomfort after birth, and the lower material costs will bring about considerable economic savings in the healthcare system (22).

### Strengths and limitations

Although adherence to the principles in clinical trials, including randomization of allocation and allocation concealment are among the strengths of this study, there are some limitations. First, we could not follow up the participants for a longer time. Perhaps with longer follow-ups, more solid evidence about sexual and marital relationships could be obtained. Second, this study was conducted in only one hospital, and two midwives conducted all deliveries and wound repairs. Future studies may investigate this topic in different hospitals and using different health providers. Another limitation is not distinguishing between 2nd degree laceration and episiotomy.

## Conclusion

The findings of our study revealed that continuous non-locking technique using vicryl rapide has a significant effect on decreasing perineal pain and improving wound healing. Furthermore, it is associated with less time for perineal repair, less suture materials, and less analgesia compared with the interrupted suturing method. These results are consistent with other studies, but given that this study was the first study to evaluate this method in Iran, we recommend that future studies be conducted in different hospitals and using different health providers in Iran to confirm the findings of this study.

## Data Availability

The original contributions presented in the study are included in the article/Supplementary Material, further inquiries can be directed to the corresponding author.
